# Assessment of the quality, oxidative status and dietary energy value of lipids used in non‐ruminant animal nutrition

**DOI:** 10.1002/jsfa.11066

**Published:** 2021-02-19

**Authors:** Alexandra L Wealleans, Karen Bierinckx, Erwin Witters, Mauro di Benedetto, Julian Wiseman

**Affiliations:** ^1^ Kemin Animal Nutrition and Health Herentals Belgium; ^2^ Department of Animal Sciences, University of Nottingham Sutton Bonnington UK

**Keywords:** lipids, apparent metabolizable energy, digestible energy, broilers, pigs

## Abstract

**BACKGROUND:**

Fats and oils represent the most concentrated source of energy available to animal nutritionists and form an expensive part of the diet. Thorough understanding of lipid quality and composition are required for efficient and precise diet formulation. Therefore, 724 samples of commercially available fats and oils were assessed for fatty acid profile, oxidation status and energetic value as per the Wiseman equation, with consideration of a correction factor *K*, which is based on the presence of the energy diluting compounds moisture, impurities and unsaponifiables.

**RESULTS:**

Energy diluting compounds were widespread across fat types and sources. Average MIU (moisture, insoluble impurities and unsaponifiable matter) presence in individual oils was 5.1–28.1 g kg^−1^. Using the adapted Wiseman equation presented in the current paper, which reflects the energy diluting potential of MIU, the calculated energy values of fats and oils is reduced by up to 46% in extreme cases compared to those predicted by the original equation. From the chemical parameters, it is clear that there is limited correlation between individual measures of oxidation, with only weak negative correlations between 2‐thiobarbituric acid (TBA) and Oxidative Stability Index (OSI) values (Spearman's *ρ* between −0.20 and −0.39) and a weak to moderate negative correlation between peroxide value (PV) and OSI (Spearman's *ρ* between −0.20 and −0.59) for certain fats and oils. A moderate to very strong positive correlation between FFA and the energy diluting compounds MIU was observed for all animal fats (Spearman's *ρ* between 0.40 and 1.00).

**CONCLUSION:**

The current report highlights the large variation in composition and quality seen in commercially available fats and oils and encourages ongoing analysis and assessment rather than reliance on published values. The results also indicate that the oxidation parameters when interpreted as separate values lack the power of inferring oil and fat quality. © 2021 The Authors. *Journal of The Science of Food and Agriculture* published by John Wiley & Sons Ltd on behalf of Society of Chemical Industry.

**ABBREVIATIONS**
AME: apparent metabolizable energy
DE: digestible energy
EDC: energy diluting compounds
FA: fatty acids
FAME: fatty acid methyl ester
FFA: free fatty acid
GC: gas chromatography
I: impurities
M: moisture
Mo: moderate
NEM: non‐elutable material
OSI: Oxidative Stability Index
PCA: principal component analysis
PUFA: poly‐unsaturated fatty acid
PV: peroxide value
S: strong
SCFA: short‐chain fatty acid
TBA: thiobarbituric acid
TBARS: thiobarbituric acid reactive substances
TG: triglyceride
U: unsaponifiables
U:S: unsaturated *versus* saturated ratio
VS: very strong
VW: very weak
W: weak

## INTRODUCTION

Fats and oils are important raw materials in non‐ruminant diets, particularly in high‐performing genotypes that require high energy concentration diets. They are of high energy content and contribute positively to diet texture, promoting feed intake and reducing wastage,^1^, ^2^ whilst also reducing dust and improving pelleting in the mill.^3^ A major problem is that they are frequently traded on the basis of name alone, rather than by assessing dietary energy content; if any data on chemical composition are provided, information is basic. The two major variables influencing the apparent metabolizable energy (AME) and digestible energy (DE) of fats and oils are glycerol content, the ratio of unsaturated to saturated (U:S) fatty acids and the free fatty acid (FFA) content;^4^, ^5^ chain length of fatty acids is of further albeit minor importance. Predicting energy on the basis of these chemical components was the subject of a seminal paper.^6^ However, it is well known that the fatty acid content of fats and oils varies within lipid source, due to environmental, genetic and processing fluctuations in the production seed or animal.^6^, ^7^ Commercially available fats and oils may deviate from published averages, though the frequency and magnitude of these deviations is not well investigated.

Another complex within fats and oils that has at best a neutral influence on dietary energy value is moisture (M), impurities (I) and unsaponifiables (U). All lipids used in the original research leading to the development of the Wiseman equation had a maximum MIU content of 20 g kg^−1^;^8^, ^9^, ^10^ the assumption that this maximum will always be the case needs verifying in commercially obtained fats and oils. Fats and oils are unstable commodities liable to polymerization and oxidation, the more so the greater the degree of unsaturation.^7^ Accordingly, various tests that determine the oxidative status of lipids have been developed over the years. Multiple options for determination of peroxides include iodometric titration, ferric ion complexes and Fourier transformed infrared technology. Secondary oxidation products can be quantified by means of spectrometry for conjugated dienes and trienes via 2‐thiobarbituric acid (TBA) value, p‐anisidine value (p‐AnV) and carbonyl value. Sensitivity towards oxidation can be determined in specific equipment such as Rancimat and oxidative stability instrument for Oxidative Stability Index (OSI).^7^ In the current report, quantification of the oxidative damage of fats and oils is assessed via peroxide value (PV) by means of iodometric titration and malondialdehyde determination through reaction with TBA via detection with ultra‐high pressure liquid chromatography assessment according to availability of equipment, solvents and general acceptance in animal feed industry. The objective of the current article is to assess further the influence of chemical composition of commercially available fats and oils used in non‐ruminant nutrition – both in terms of oxidation, fatty acid profile and the presence of MIU – on their dietary energy value with a view to revising the equation published by Wiseman *et al*.^8^

## MATERIALS AND METHODS

Lipid samples were collected from commercial animal feed production facilities across Europe, Russia, Middle East and North Africa between 2013 and 2019. No historic record with respect to handling, storage, stabilization with antioxidants (natural or synthetic), or treatment prior to reception of the samples is present; lipids subjected to excessive processing or suboptimal handling might be present in this dataset. All samples were analysed for chemical composition within 2 weeks of receipt. Before analysis, samples were kept refrigerated at 277 K in glass containers. Ten major lipid sources were selected for in depth analysis, including six vegetable oils and four animal fats, comprising 724 field samples. The selected samples were all considered triglycerides (TGs); other oil types – technical fats and by‐products – including calcium soapstocks, fatty acid distillates and blends were not included in the final selection of samples.

The chemical complex representing energy‐diluting compounds is assessed by the analysis of its largest contributors MIU. Together with breakdown products as well as polymerization products, MIU are commonly described as a component of non‐elutable material (NEM^9^). A more detailed analysis of compounds that constitute NEM is outside of the scope of the current study. However, it is noted that the energy diluting compounds as represented by MIU is an underestimation of the real loss of nutritional energy.

### Chemical characteristics of fats and oils used in animal nutrition

#### Peroxide values

The method used is based upon methods AOAC 965.33 and AOCS CD 8‐53. A potentiometric titrator (Kyoto Electronics, Kyoto, Japan) equipped with a platinum electrode and a reference electrode was used to titrate the samples. Briefly, 500 mg of homogenized sample was dissolved in 30 cm^3^ methylene chloride/acetic acid (2:3) (Acros Chemicals, Geel, Belgium). After complete dissolution, 0.5 cm^3^ saturated potassium iodide (Acros Chemicals) was added and allowed to react for 1 min. Next, 30 cm^3^ milliQ water (Millipore, Bedford, MA, USA) was added and the mixture was titrated with 1 mmol dm^−3^ sodium thiosulphate solution immediately with a potentiometric titrator. Sodium thiosulphate solution was prepared by diluting Titrisol (Merck Cat. No. 1.09909.0001) according to the manufacturer's instructions. For all oxidative parameters, samples were categorized by level of severity of oxidation, summarized in Table 1.^10^ These categories reflect practical classifications used in the field to assess the oxidative status of lipids for use in animal nutrition.

**Table 1:** Ranges assigned to oxidative quality categories for the fats and oils assessed in the dataset. These ranges allow nutritionists to give broad assessments of the oxidative quality of the fat sample they are evaluating^9^, ^11^

	Peroxide value	2‐Thiobarbituric acid value (ppm)	
	(meq kg^−1^)	Non‐fish oils	Fish oils
No oxidation	<5	<0.5	<5
First signs of oxidation	5–10	0.5–1	5–10
Oxidation	10–20	1–2	10–20
Strong oxidation	>20	>2	>20

#### Thiobarbituric acid values

Measurement of TBA values was chosen as the analytical parameter measuring secondary oxidation because of its ease of use and limited consumption of toxic solvents during the assessment. A homogenous sample of 5 g was extracted with 50 cm^3^ trichloroacetic acid (TCA, 50 mg cm^−3^) for 2700 s. After centrifugation, a 2 cm^3^ aliquot of clear supernatant was mixed with 2 cm^3^ of TBA (2 mg cm^−3^) in a capped glass test tube and kept at 373.15 K for 1800 s. Next, malondialdehyde was determined by injecting 10 mm^3^ on a chromatography system (UltiMate 3000, Thermo Scientific, Waltham, MA, USA) equipped with an auto sampler, a reversed phase column (1.7 μm C18 100A, 50 mm × 2.1 mm, 100 Å, Kinetex, Phenomenex, Utrecht, the Netherlands) and a UV‐visible detector set at *λ* = 532 nm. Isocratic runs were kept at a constant flow rate of 8.3 mm^3^ s^−1^. The eluent, a buffer solution containing acetic acid (5 mg cm^−3^) and triethylamine (8 mg·cm^−3^, pH 7), was set at an isocratic flow of 8.3 mm^3^·s^−1^. All chemicals were supplied by Acros Chemicals. Standard solutions were prepared by diluting 1,1,3,3‐tetraethoxy propane (0.2 mg cm^−3^ of a 97% stock solution) with TCA (50 mg cm^−3^) covering the range of 0.2 to 20 ppm malondialdehyde.

#### Oxidative stability instrument

By means of an accelerated test, the oxidative stability of the treated lipids was assessed with a specific oxidative stability instrument (Omnion Inc., Rockland MA, USA) according to the instructions of the manufacturer. Briefly, 10 g of each lipid sample was placed in a glass tube, heated at 373.15 K and continuously flushed with air at a flow of 2.24 to 2.72 cm^3^ s^−1^. Next, a conductivity cell, with 50 cm^3^ milliQ water (Millipore) captured the volatile oxidized breakdown products of the fats and oils via airflow from the glass cell. The formation of secondary oxidation products leads to an increase in the conductivity of the milliQ water and is monitored over time. When the oxidation process enters in its propagation phase, a sharp increase in conductivity is observed. This is automatically calculated and reported by the OSI software. The longer the induction time, OSI, the more stable a fat or oil is towards oxidation.

#### Free fatty acid content

The protocol of the method used to determine the FFA content is based upon AOCS method Ca 5a‐40. Ethanol (Acros Chemicals) was heated to approximately 353 K in a water bath. A few drops of phenolphthalein (VWR, Leuven, Belgium) solution were added and sodium hydroxide (Acros Chemicals) (0.1 mol dm^−3^) solution was added drop wise until a faint pink colour persisted. Equipment: An automatic titrator equipped with a glass electrode and a reference electrode for non‐aqueous phase (Kyoto Electronics) was used to titrate the samples. Liquid oil or molten fat was homogenized, and 1 g was weighed into a 1 dm^3^ beaker. A total of 50 cm^3^ neutralized ethanol (353 K) was added and titrated immediately with sodium hydroxide (0.1 mol dm^−3^) solution with the automatic titrator under constant stirring. The FFA value is expressed relative to equivalents of oleic acid [molecular weight (MW) = 2 824 614 g mol^−1^].

#### Ratio of unsaturated over saturated fatty acid content

The method used for determination of the fatty acid profile of fats and oils is based upon the methodology described in AOAC 996.06 [Fat (total, saturated, and unsaturated) in Foods – Hydrolytic Extraction Gas Chromatographic Method). The degree of saturation was described as the ratio of unsaturated over saturated (U:S) fatty acids. The fatty acid composition of both the TG fraction and the FFA fraction of a lipid was determined indirectly with gas chromatography (GC) analysis by (trans‐)esterification reaction resulting in fatty acid methyl esters (FAMEs). Initially, 40 mg homogenized fat was brought in a glass pressure tube and dissolved in 2 cm^3^ heptane (VWR). A total of 2 cm^3^ of a methanolic sodium hydroxide (VWR) solution (0.5 mol dm^−3^) was added after which the pressure tube was closed and placed for 1 h at 352 K in a water bath. After cooling down to room temperature, 2 cm^3^ of methanolic boron trifluoride (20%) (VWR) was added to the tube. The tube was placed back into the water bath at 352 K for half an hour. After cooling down, 1 mm^3^ of the organic layer was dissolved in 1 cm^3^ heptane and 100 μm^3^ split less injected into a gas chromatograph system (Thermo Scientific) with flame ionization detection (523 K, airflow: 58 cm^3^ s^−1^, H_2_ flow: 5.8 cm^3^ s^−1^, nitrogen make‐up flow: 5 cm^3^ s^−1^). Separation of the FAMEs was done on a 100 m Restek (Rt‐2560, 0.25 mm ID, 0.20 μm, Bellefonte, PA, USA) column using the following settings for the oven temperature: 343 K for 120 s, ramp 1: 0.25 K s^−1^ till 408 K and holding 180 s, ramp 2: 0.075 K s^−1^ till 451 K and holding 180 s, ramp 3: 0.33 K s^−1^ till 513 K and holding 240 s. The inlet temperature was kept at 533 K. The peak areas were used to calculate the ratio of unsaturated over saturated fatty acids. The fatty acids with a short carbon chain length (i.e. ≤ C14:0), are regarded as unsaturated compounds irrespective of their degree of saturation.

#### Assessment of moisture, impurities and unsaponifiables

Moisture in oil samples was analysed as per the experimental protocol ISO8534:2012 [animal vegetables fats and oils – determination of water content – Karl Fisher method (pyridine free)]. Reagents, equipment and experimental procedure for the assessment of impurities and unsaponifiables in oil samples were conducted according to the protocol of ISO663 (animal and vegetable fats and oils – determination of insoluble impurities – Gravimetric method) and ISO3596:2012 (animal and vegetable fats and oils – determination of unsaponifiable matter – method using diethyl ether extraction), respectively.

For all analytical parameters, single measurement data were acquired. For values below the lower limit of detection, data imputation (square root of the detection limit) was done in order to allow statistical analysis of the dataset.

#### Calculation of energy value of fats and oils

The energy value of fats and oils is calculated by the Wiseman equation (Eqn (1)^8^). Energy is determined by the content of FFA and the U:S ratio of the fatty acids. The constants *A*, *B*, *C* and *D* differ between species and the age of the animal, and are defined in the original paper.^8^

Equation (1) represents the estimation of AME (poultry) or DE (pigs) by means of the Wiseman equation.

(1) $$\text{Energy, MJ/kg}\;=A+B\;\times \;\mathrm{FFA}+C\;\times \;e^{D\left(\frac{U}{S}\right)}$$

### Statistical assessments of the dataset

Statistics were performed using the XLSTAT plugin for Excel.^12^ Normality testing (Shapiro–Wilk test, *α* = 0.05) reveals non‐normality for the majority of the data. Outliers in the data set are identified by means of the modified *Z*‐score with the median (*x̃*) absolute deviation (MAD = median{|xi − *x̃*|}) as the estimator. For the discussion of the chemical characteristics a censored dataset where the upper extreme values are removed was used. Their frequency was low and where possible their causes are explained in the Results and Discussion sections.

Spearman type correlation tests are used to identify correlations between the characteristics in a given lipid. A Spearman correlation coefficient between 0 and 0.19 reflect a very weak (VW) correlation, between 0.20 and 0.39 is a weak (W) correlation, between 0.40 and 0.59 is a moderate (Mo) correlation, between 0.60 and 0.80 is considered a strong (S) correlation and between 0.80 and 1 indicates a very strong (VS) correlation. The significant correlations are listed (Tables 2 and 3).

**Table 2:** Correlation matrix for chemical characteristics from vegetable oils

Variable	PV	OSI	FFA	MIU	U:S	SFA	MUFA	PUFA
TBA	Soybean (−0.146)	Soybean (−0.156)	Corn (−0.537) Soybean (0.223)	—	—	Rapeseed (0.492)	—	—
PV		Soybean (−0.199) Sunflower (−0.425)	Rapeseed (0.611)	Soybean (0.151)	Corn (0.668)	Corn (−0.534)	Soybean (0.335)	Soybean (−0.251)
OSI			—	—	Soybean (0.146)	—	Sunflower (0.321)	Sunflower (−0.276)
FFA				—	—	Soybean (−0.131)	Corn (−0.743) Soybean (0.277)	Corn (0.625) Palm (0.451) Soybean (−0.165)
MIU					Corn (S)	—	Soybean (0.161)	Soybean (−0.509)
U:S						Corn (−0.938) Linseed (−1.000) Palm (−0.982) Rapeseed (−0.998) Soybean (−0.999) Sunflower (−0.999)	Linseed (−0.744) Palm (0.802) Rapeseed (0.598) Sunflower (0.359)	Linseed (0.861) Palm (0.779) Soybean (0.493)
SFA							Linseed (0.744) Palm (−0.800) Soybean (0.161) Sunflower (−0.332)	Linseed (−0.861) Palm (−0.647) Soybean (−0.509)
MUFA								Corn (−0.809) Linseed (−0.963) Rapeseed (−0.909) Soybean (−0.832) Sunflower (−0.984)

Only significant (*P* < 0.05) correlations are shown.

Spearman correlation coeefficient between 0.00 and 0.19: very weak (VW), 0.20 and 0.39: weak (W), 0.40 and 0.59: moderate (Mo), 0.60 and 0.79: strong (S), 0.80 and 1.0: very strong (VS).

TBA, 2‐thiobarbituric acid; PV, peroxide value; OSI, Oxidative Stability Index; FFA, free fatty acid; MIU, moisture, impurities and unsaponifiables; U;S, unsaturated/saturated ratio; SFA, saturated fatty acid; MUFA, mono‐unsaturated fatty acid; PUFA, poly‐unsaturated fatty acid.

**Table 3:** Correlation matrix for chemical characteristics from animal fats

Variable	PV	OSI	FFA	MIU	U:S	SFA	MUFA	PUFA
TBA	—	Lard (−0.254) Poultry (−0.335)	Lard (0.361)	Lard (0.500)	Poultry (−0.250)	Poultry (0.243)	Lard (0.212)	Poultry (−0.246)
PV		—	Lard (−0.245)	Poultry (−0.321)	—	—	Lard (0.177) Poultry (−0.252)	—
OSI			Lard (−0.198) Poultry (−0.330)	Lard (−0.186)	Poultry (0.272)	Poultry (−0.248)	Fish (0.426)	Poultry (0.223)
FFA				Fish (0.401) Lard (0.778) Poultry (0.668) Tallow (0.733)	Poultry (−0.296)	Poultry (0.314)	Lard (−0.198 Poultry (0.199)	Poultry (−0.323) Tallow (0.393)
MIU					Poultry (−0.279)	Poultry (0.266)	Poultry (0.288)	Poultry (−0.323) Tallow (0.538)
U:S						Fish (−0.998) Lard (−0.999) Poultry (−0.991) Tallow (−0.997)	Fish (0.761) Lard (0.731) Tallow (0.924)	Lard (0.639) Poultry (0.806) Tallow (0.399)
SFA							Fish (−0.690) Lard (−0.738) Tallow (−0.925)	Lard (−0.630) Poultry (−0.816)
MUFA								Poultry (−0.484)

Only significant (*P* < 0.05) correlations are shown.

Spearman correlation coeefficient between 0.00 and 0.19: very weak (VW), 0.20 and 0.39: weak (W), 0.40 and 0.59: moderate (Mo), 0.60 and 0.79: strong (S), 0.80 and 1.0: very strong (VS).

TBA, 2‐thiobarbituric acid; PV, peroxide value; OSI, Oxidative Stability Index; FFA, free fatty acid; MIU, moisture, impurities and unsaponifiables; U;S, unsaturated/saturated ratio; SFA, saturated fatty acid; MUFA, mono‐unsaturated fatty acid; PUFA, poly‐unsaturated fatty acid.

**Figure 1:**
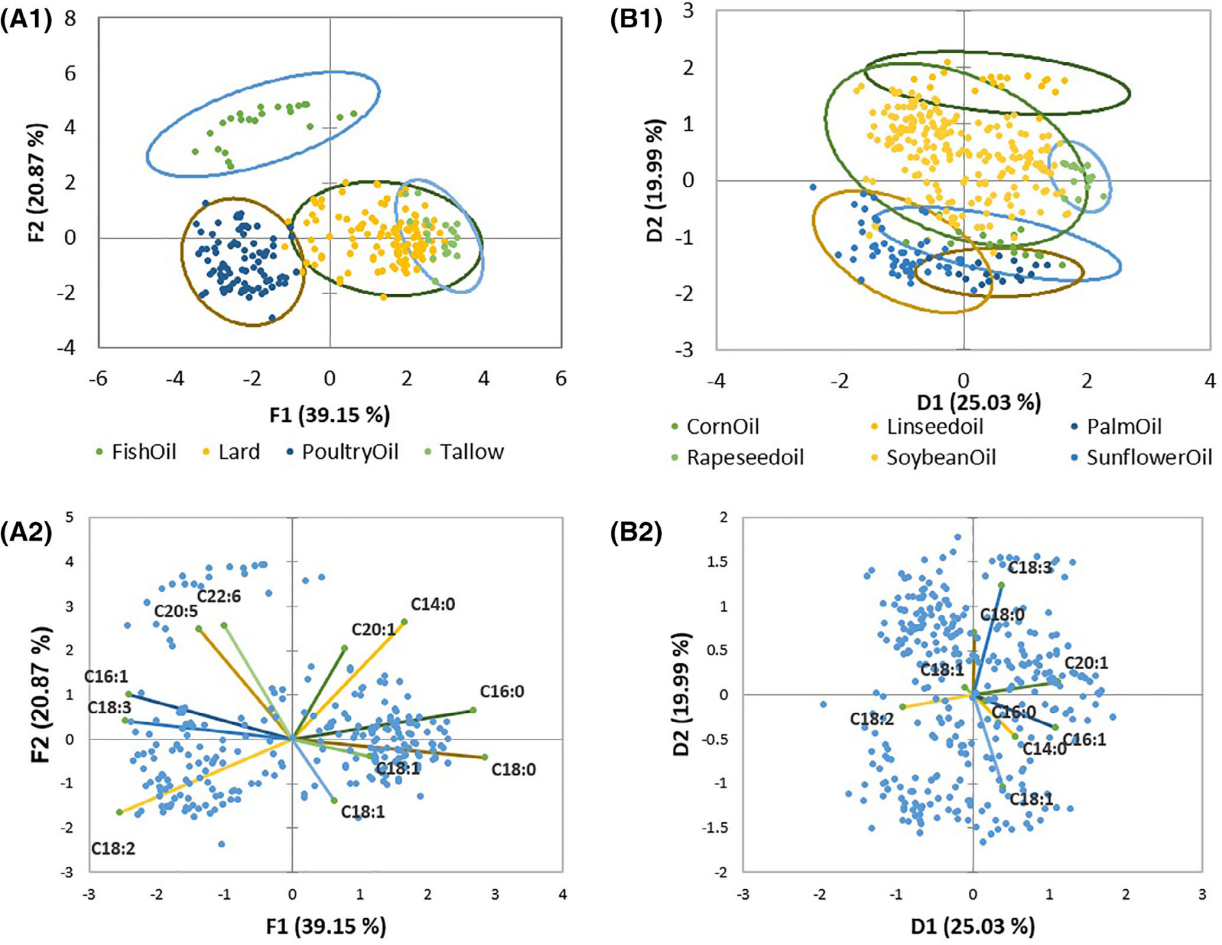
Principal component analysis of 11 predominant fatty acids for animal fats (observation plot pane A1, biplot pane A2) and nine predominant fatty acids for plant oils (observation plot pane B1, biplot pane B2). Ellipses indicate the 95% confidence interval.

Spearman type principal component analysis (PCA) of the predominant fatty acids obtained by the FAME profiles for plant and animal samples is presented in Fig. 1. A varimax rotation followed by a Kaiser normalization was performed (factors *n* = 3) to produce the symmetric type of biplots.

## RESULTS

### Oxidative status of commercial fats and oils

**Figure 2:**
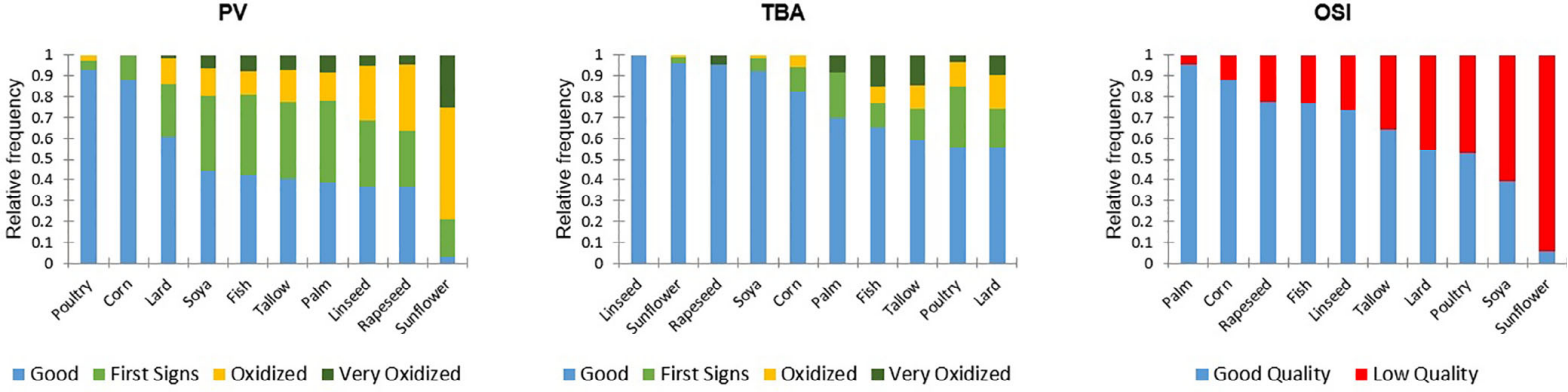
Proportion of samples from each individual oil type displaying limited (‘good’), initial, or substantial signs of oxidation according to the three tests: PV, TBA and OSI.

Lipids in the current dataset were evaluated for severity in oxidation based on PV and TBA values, as shown in Tables 4, 5, 6. The overall severity of primary oxidation products (PV) was significantly different between oils (Fig. 2). Sunflower oil had the highest median PV value (15 meq kg^−1^), with only 0.03 of the samples having a good PV. Except for corn oil (*x̅* = 2.6 meq kg^−1^), lard (*x̅* = 4.6 meq kg^−1^) and notably poultry fat (*x̅* = 2.1 meq kg^−1^) for which a good PV‐quality was recorded for 0.88, 0.61 and 0.93 of all samples tested. All other oils had PVs that indicated oxidation (≥ 5 ppm) for the majority of their samples tested.

**Table 4:** Oxidative, nutritional and energy‐related parameters of vegetable (soybean, rapeseed and sunflower) oils obtained from commercial animal nutrition sources

		Soybean oil				Rapeseed oil				Sunflower oil			
		Minimum	Maximum	Average	*σ*	Minimum	Maximum	Average	*∑*	Minimum	Maximum	Average	*σ*
*n*		245				219				72			
*Oxidative parameters*													
PV (meq kg^‐1^)		0.05	17.97	6.10	3.70	1.00	19.67	8.23	5.91	1.40	31.96	15.45	6.16
TBA (ppm)		0.10	1.40	0.19	0.21	0.10	3.10	0.27	0.64	0.01	1.02	0.13	0.14
OSI 100°C (h)		2.90	24.00	12.79	3.40	2.75	35.65	17.78	9.03	4.10	12.70	8.96	1.64
*Nutritional parameters*													
FFA (% C18:1)		0.00	2.09	0.60	0.47	0.00	1.40	0.59	0.46	0.03	1.53	0.69	0.33
MUFA		18.95	31.96	25.86	2.16	61.82	70.30	67.35	2.41	18.04	39.35	30.73	4.81
PUFA		48.36	63.35	56.72	2.45	23.08	30.57	25.85	2.07	49.79	71.15	58.40	4.54
*Fatty acid profile*													
C14:0		0.00	0.21	0.09	0.05	0.00	0.10	0.05	0.02	0.00	0.16	0.07	0.04
C16:0		9.88	14.25	12.07	0.71	4.07	5.65	4.85	0.31	5.93	8.01	7.03	0.46
C16:1		0.00	0.21	0.09	0.05	0.10	0.25	0.15	0.04	0.00	0.17	0.08	0.04
C18:0		3.30	7.03	5.02	0.85	1.31	2.32	1.68	0.26	2.54	4.69	3.69	0.40
C18:1		0.00	2.01	0.05	0.16	0.00	0.11	0.02	0.03	0.00	0.17	0.02	0.03
C18:2		19.31	31.35	25.52	2.09	60.90	69.23	66.09	2.51	18.04	39.10	30.50	4.80
C18:3		0.00	2.01	0.05	0.16	0.00	0.11	0.02	0.03	0.00	0.17	0.02	0.03
U:S ratio		3.63	5.94	4.78	0.36	11.49	16.15	14.31	1.04	6.71	9.76	8.24	0.58
*Energy diluting factors*													
Moisture (%)		0.01	0.19	0.08	0.03	0.03	0.14	0.07	0.03	0.01	0.14	0.07	0.02
Impurities (%)		0.00	0.07	0.02	0.01	0.00	0.04	0.01	0.01	0.01	0.07	0.02	0.02
Unsaponifiables (%)		0.30	1.12	0.70	0.13	0.74	1.30	1.00	0.13	0.40	1.14	0.77	0.13
Total MIU (%)		0.39	1.30	0.81	0.15	0.83	1.39	1.10	0.14	0.45	1.52	0.90	0.19
*Energy value (kcal.kg* ^*−1*^ *)*													
AME young poultry		8578.93	8984.18	8761.24	71.18	9042.35	9100.95	9081.59	15.10	8957.82	9051.73	9015.97	21.55
AME old poultry		8889.13	9173.44	9012.98	47.63	9252.17	9316.38	9297.33	15.84	9169.40	9268.48	9223.43	20.03
DE young pigs		8778.64	8988.54	8874.12	37.11	9018.66	9048.96	9038.64	7.93	8973.53	9023.89	9005.55	11.19
DE old pigs		8749.97	8804.54	8781.07	10.62	8796.24	8812.93	8805.77	5.56	8794.57	8811.43	8802.93	4.02
*Energy value including MIU (kcal kg* ^*‐1*^ *)*													
AME young poultry		8421.97	8919.65	8686.09	82.37	8936.19	9020.75	8982.01	22.27	8824.39	8985.11	8930.82	33.66
AME old poultry		8739.93	9128.69	8941.04	61.86	9143.55	9231.91	9195.38	23.01	9031.57	9196.46	9136.12	32.74
DE old pigs		8661.79	8925.57	8801.67	45.81	8904.32	8970.82	8939.53	16.81	8838.59	8960.56	8921.71	25.29
DE young pigs		0.39	1.30	0.81	0.15	0.83	1.39	1.10	0.14	0.45	1.52	0.90	0.19

TBA, 2‐thiobarbituric acid; PV, peroxide value; OSI, Oxidative Stability Index; FFA, free fatty acid; MIU, moisture, impurities and unsaponifiables; U;S, unsaturated/saturated ratio; MUFA, mono‐unsaturated fatty acid; PUFA, poly‐unsaturated fatty acid; AME, apparent metabolizable energy; DE, digestible energy.

**Table 5:** Oxidative, nutritional and energy‐related parameters of vegetable (corn, linseed and palm) oils obtained from commercial animal nutrition sources

		Corn Oil				Linseed Oil				Palm Oil			
		Minimum	Maximum	Average	*σ*	Minimum	Maximum	Average	*σ*	Minimum	Maximum	Average	*σ*
*n*		17				20				20			
*Oxidative parameters*													
PV (meq kg^−1^)		1.02	6.11	2.58	1.52	2.27	21.62	8.65	5.57	1.00	18.06	5.81	4.28
TBA (ppm)		0.10	1.20	0.24	0.32	0.10	0.41	0.14	0.08	0.10	0.71	0.29	0.21
OSI 100°C (h)		0.00	34.00	19.16	9.67	2.80	72.00	17.54	15.55	0.00	72.00	29.18	24.26
*Nutritional parameters*													
FFA (% C18:1)		10.77	14.35	11.94	1.08	0.00	6.03	1.02	1.24	0.00	6.97	3.52	2.50
MUFA		28.48	40.22	32.21	3.13	18.83	40.29	23.48	4.85	36.71	42.05	39.75	1.31
PUFA		43.95	55.30	51.16	2.69	9.65	71.32	63.48	13.23	5.30	11.29	8.69	1.38
*Fatty acid profile*													
C14:0		0.00	0.24	0.08	0.07	0.00	1.04	0.09	0.23	0.64	1.30	0.96	0.22
C16:0		9.24	16.04	13.47	2.01	5.87	44.49	8.51	8.49	42.11	51.29	45.70	2.35
C16:1		0.00	0.20	0.11	0.06	0.00	0.28	0.11	0.08	0.00	0.26	0.12	0.07
C18:0		1.52	3.49	2.39	0.46	3.43	5.94	4.45	0.79	3.20	6.04	4.57	0.68
C18:1		0.00	2.33	0.34	0.72	0.00	0.47	0.03	0.11	0.00	0.34	0.04	0.09
C18:2		27.84	44.91	32.25	4.32	18.59	39.45	23.05	4.69	36.55	41.78	39.25	1.38
C18:3		0.00	2.33	0.34	0.72	0.00	0.47	0.03	0.11	0.00	0.34	0.04	0.09
U:S ratio		4.33	6.12	5.06	0.55	1.00	9.39	7.77	1.87	0.77	1.09	0.95	0.08
*Energy diluting factors*													
Moisture (%)		0.01	0.76	0.30	0.22	0.04	0.12	0.08	0.02	0.01	0.12	0.07	0.03
Impurities (%)		0.01	0.34	0.09	0.09	0.01	0.10	0.07	0.02	0.01	0.11	0.04	0.03
Unsaponifiables (%)		1.14	3.01	2.38	0.60	0.33	1.09	0.89	0.20	0.27	0.54	0.38	0.07
Total MIU (%)		0.03	4.07	2.54	1.09	0.44	1.25	1.03	0.21	0.33	0.72	0.51	0.10
*Energy value (kcal.kg* ^*−1*^ *)*													
AME young poultry		8415.22	8879.00	8590.90	151.89	6768.70	9062.98	8919.58	506.94	6566.95	6914.99	6766.07	90.04
AME old poultry		8781.66	9105.93	8904.45	104.98	7878.89	9271.79	9172.34	314.21	7697.86	7972.34	7877.17	61.66
DE young pigs		8683.77	8905.97	8754.84	58.13	7809.77	9029.75	8953.85	269.61	7700.95	7890.67	7809.10	48.82
DE old pigs		8611.37	8659.59	8645.60	13.09	8035.66	8810.92	8764.49	171.60	7915.70	8117.28	8032.30	52.64
*Energy value including MIU (kcal.kg* ^*−1*^ *)*													
AME young poultry		8131.29	8750.93	8367.07	206.99	6738.85	8983.59	8823.76	505.39	6339.30	6883.39	6677.98	136.41
AME old poultry		8415.25	8967.94	8666.20	178.00	7844.14	9187.87	9077.40	299.56	7679.64	7935.91	7834.04	61.52
DE old pigs		8269.99	8794.41	8540.49	159.56	7775.33	8950.65	8859.41	262.93	7604.61	7854.61	7756.98	61.19
DE young pigs		0.03	4.07	2.54	1.09	0.44	1.25	1.03	0.21	0.33	0.72	0.51	0.10

TBA, 2‐thiobarbituric acid; PV, peroxide value; OSI, Oxidative Stability Index; FFA, free fatty acid; MIU, moisture, impurities and unsaponifiables; U;S, unsaturated/saturated ratio; MUFA, mono‐unsaturated fatty acid; PUFA, poly‐unsaturated fatty acid; AME, apparent metabolizable energy; DE, digestible energy.

**Table 6:** Oxidative, nutritional and energy‐related parameters of animal fats obtained from commercial animal nutrition sources

		Fish oil				Lard				Tallow				Poultry fat			
		Minimum	Maximum	Average	*σ*	Minimum	Maximum	Average	*σ*	Minimum	Maximum	Average	*σ*	Minimum	Maximum	Average	*σ*
*n*		26				158				27				113			
*Oxidative parameters*																	
PV (meq kg^−1^)		1.00	13.48	6.39	3.26	1.00	13.27	4.63	2.98	1.00	13.27	5.46	3.33	0.39	5.66	2.16	1.05
TBA (ppm)		0.10	12.60	3.50	3.35	0.10	1.74	0.49	0.46	0.10	1.50	0.44	0.46	0.10	1.93	0.50	0.41
OSI 100°C (h)		0.35	45.85	12.98	13.83	0.00	39.60	8.44	10.08	0.00	42.05	9.79	11.76	0.00	59.50	13.63	15.65
*Nutritional parameters*																	
FFA (% oleic acid)		0.01	9.13	4.83	2.36	0.03	22.75	8.74	6.57	0.38	16.07	6.57	4.63	0.72	23.29	9.37	4.26
MUFA		17.40	58.96	38.33	9.87	38.51	51.56	44.93	2.77	31.35	51.36	42.35	4.77	38.50	49.71	43.96	2.15
PUFA		15.85	33.30	23.72	3.97	6.43	16.52	9.59	2.42	0.99	6.54	4.14	1.81	17.60	34.17	23.22	3.85
*Fatty acid profile*																	
C14:0		3.02	14.27	7.37	3.08	0.47	2.69	1.60	0.38	1.43	3.41	2.34	0.54	0.32	1.65	0.85	0.32
C16:0		11.99	31.31	23.53	5.72	23.52	31.55	27.16	1.55	25.99	32.26	28.51	1.70	20.36	27.43	23.90	1.36
C16:1		3.54	12.60	7.59	2.87	1.50	5.36	2.78	0.70	1.25	3.54	2.54	0.51	2.66	5.58	4.04	0.51
C18:0		2.58	7.59	5.04	1.38	7.01	23.03	15.86	3.15	10.78	32.08	21.32	4.38	4.94	10.28	7.65	1.37
C18:1		0.00	0.35	0.10	0.10	0.00	1.39	0.48	0.40	0.00	2.71	0.89	0.73	0.00	0.57	0.22	0.13
C18:2		0.59	51.24	26.81	11.28	34.90	46.88	40.48	2.37	29.32	47.73	38.94	3.68	34.87	44.81	39.23	1.93
C18:3		0.00	0.35	0.10	0.10	0.00	1.39	0.48	0.40	0.00	2.71	0.89	0.73	0.00	0.57	0.22	0.13
U:S ratio		0.98	3.28	1.66	0.53	0.83	1.84	1.25	0.22	0.26	1.13	0.86	0.21	1.54	2.77	2.05	0.27
*Energy diluting factors*																	
Moisture (%)		0.05	0.36	0.13	0.08	0.01	0.82	0.23	0.18	0.03	0.41	0.16	0.11	0.02	0.65	0.24	0.14
Impurities (%)		0.00	0.11	0.03	0.03	0.01	0.61	0.14	0.14	0.01	0.26	0.07	0.07	0.00	0.37	0.11	0.08
Unsaponifiables (%)		0.49	1.76	1.05	0.34	0.01	8.30	2.00	1.78	0.20	2.90	1.04	0.80	0.06	4.50	2.38	0.94
Total MIU (%)		0.63	2.57	1.38	0.52	0.03	10.06	2.63	2.25	0.27	4.95	1.81	1.46	0.29	5.18	2.81	0.99
*Energy value (kcal.kg* ^*−1*^ *)*																	
AME young poultry		6956.39	8716.99	7670.34	476.60	6206.57	7634.80	6987.58	271.94	6315.56	7111.06	6684.04	190.94	6970.98	8192.45	7634.48	228.70
AME old poultry		7970.98	8828.17	8332.12	242.29	7540.90	8335.43	7978.90	152.12	7636.33	8030.96	7828.25	102.40	7912.39	8647.07	8314.79	134.62
DE young pigs		7910.84	8850.39	8293.55	253.74	7500.69	8276.87	7926.56	147.76	7565.74	7991.53	7763.81	103.17	7910.92	8573.84	8272.92	123.69
DE old pigs		8139.44	8766.12	8444.00	164.66	7715.67	8467.11	8149.01	142.57	7749.33	8209.72	7982.58	111.89	8117.16	8655.23	8434.13	98.91
*Energy value including MIU (kcal.kg* ^*−1*^ *)*																	
AME young poultry		6837.71	8400.99	7520.43	423.21	5721.21	7624.26	6802.87	347.43	5785.08	7011.15	6480.00	275.62	6727.97	8016.22	7410.46	272.01
AME old poultry		7835.00	8738.13	8214.33	235.11	6914.63	8323.93	7771.92	275.02	7170.50	7987.12	7642.50	192.16	7518.97	8528.13	8070.50	195.91
DE young pigs		7775.88	8638.44	8154.35	224.43	6869.15	8265.45	7720.36	264.39	7100.83	7911.53	7567.11	193.94	7521.16	8460.81	8029.77	184.23
DE old pigs		0.63	2.57	1.38	0.52	0.03	10.06	2.63	2.25	0.27	4.95	1.81	1.46	0.29	5.18	2.81	0.99

TBA, 2‐thiobarbituric acid; PV, peroxide value; OSI, Oxidative Stability Index; FFA, free fatty acid; MIU, moisture, impurities and unsaponifiables; U;S, unsaturated/saturated ratio; MUFA, mono‐unsaturated fatty acid; PUFA, poly‐unsaturated fatty acid; AME, apparent metabolizable energy; DE, digestible energy.

**Figure 3:**
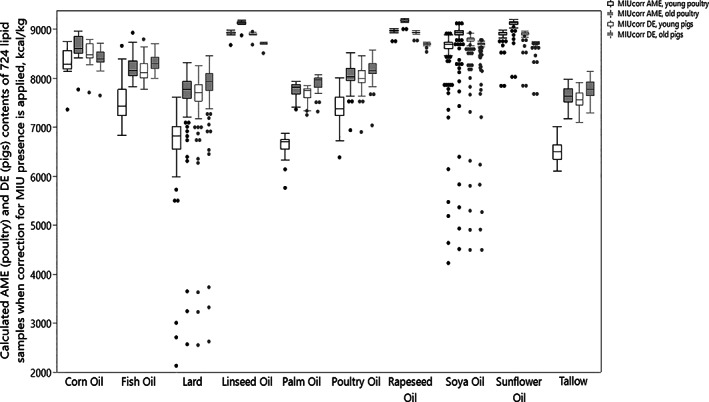
Comparison of the energy value for individual samples based on both the original Wiseman equation and the modified equation including MIU as energy diluting component of soybean oil, sunflower oil, rapeseed oil, tallow, lard and poultry fats tested.

Secondary oxidation products, TBA, were detected in 0.11, 0.00 and 0.07 of the samples tested for respectively soybean, rapeseed and sunflower oils. The average result for oxidation is between 0.5 and 2 ppm; only 0.04 of samples showed strong oxidation (> 2 ppm). The majority of samples showed good TBA value for all individual oils (Tables 2 and 3, Fig. 3). Most samples showed oxidation in its initial phase (0.76 of all samples indicating no oxidation, 0.13 demonstrating the first signs of oxidation), with the exception of fish oil and tallow where more evolved oxidation was registered in 0.23 and 0.26 of samples.

Correlation analysis reveals few significant relationships between the separate measures of oxidation, as shown in Tables 2 and 3. There are few, significant but weak correlations between PV and TBA or OSI throughout the dataset, indicating that primary and secondary oxidation reactions occur separately. This is in contrast with earlier described research by Frankel^13^ and observations made in laboratory conditions where primary and secondary oxidation occur sequentially.

### Nutritional value and energy diluting factors of commercially obtained fats and oils

The PCA presented in Fig. 1 is done on predominant fatty acids present in animal fats and plant oils. Figure 1(A1 and B1) show a clear range of the individual groups based upon their fatty acid composition of the lipids, origin of the sample and number of samples analysed. Figure 1(A2 and B2) show the positioning of the different fatty acids present in the tested fats and oils amongst each other. This relative spreading is the basis for the differentiation between the lipid types in the PCA. Large spreading between the groups is observed between the various animal fats. Fish oil is clearly separated from the other animal fats due to the presence of typical fish oil C20:5 and C22:6 fatty acids. Poultry fat contains higher values of C18:2 compared to the others and therefore splits from the other fats. Tallow and lard are more closely related in terms of fatty acid composition, resulting in overlapping ellipses. For plant oils, more overlap between the groups is observed. The natural presence of different fatty acids is less unique for a specific plant oil when compared to animal fats. Though differentiation is observed between the different groups and in some cases – for example linseed – are more pronouncedly separated from the others. While it is well known that lipids from different biological sources have varying fatty acid composition,^14^, ^15^, ^16^ the data presented in the current study indicate that there is often a large overlap in composition between lipid sources. Where local market regulations and commercial pressures discourage or encourage the use of particular raw materials in animal diets, it is important for nutritionists to be able to conclusively identify raw material origins; this overlap means that fatty acid composition alone may not be sufficient to distinguish chemically, for example, between sunflower and corn oils or between soybean and corn oils, as shown in Fig. 1(B1).

Tables 4, 5, 6 demonstrates a large variation in energy values within an individual lipid source. For soybean oil, values range between 7900 and 8980 kcal kg^−1^ (calculated for young poultry; 238.85 kcal are equivalent to 1 MJ), with a standard deviation of 150 kcal kg^−1^, when calculated with the original Wiseman equation. Very low variation in calculated energy values was seen in linseed, rapeseed, and sunflower oils, with standard deviations of 27, 42 and 31 kcal kg^−1^. There was, however, very large variation in energy value for all the animal fats and palm oil.

These variations in energy values are themselves due to the large variations in percentage of FFA as well as the variation in composition of the oils. Indeed, and except for fish oil where C20:5 and C22:6 are diagnostic, the contribution of the individual components account for a rather poor resolution when describing the oils by two principal components (Fig. 1). For soybean oil, the published U:S ratio is 5.2,^17^ while in this dataset samples ranged from 3.6 to 5.9, with an average of 4.8. The tighter ranges seen for some oils may relate to the relatively low number of samples analysed for these oils, though this is not the case for lard or poultry fat. These oils are highly processed, and likely to be more standardized during production than vegetable oils. Maximum levels of FFA as g kg^−1^ of oleic acid reached 230 (poultry fat), with similar maxima in other oils (230 lard, 160 tallow, 140 corn oil, 90 fish oil).

Maximum values of 540 g kg^−1^ moisture (lard) and 160 g kg^−1^ impurities (soybean oil) were recorded; these observations – though authentic data – were extreme cases and therefore considered outliers for the assessment of correlation. The unsaponifiable fraction is the largest contributor to MIU in general, especially in soybean oil, sunflower oil, lard and poultry fat, where extreme values of 452, 12, 193 and 164 g kg^−1^ were found, respectively. The unsaponifiable fraction also drives the majority of correlations between other parameters and total MIU, with moisture and unsaponifiable levels uncorrelated, but with significant positive correlations between moisture and impurities.

Since there is no energy contribution of MIU, a correction is needed for the accurate calculation of the nutritional value of lipids. This correction factor is presented by Eqn (2) and is a dilution of the energy value calculated by Eqn (1).

Equation 2 represents the extended Wiseman equation containing MIU (%) correction

(2) $$\text{Energy}=\left[A+B\times \mathrm{FFA}+C\times e^{D\left(\frac{U}{S}\right)}\right]K$$

where *K* is a constant representing the correction factor for energy diluting compounds, calculated as 1 – energy diluting compounds/100.

Inclusion of MIU in the nutritional value estimation leads to an increase in energy variation; as shown in Fig. 3. When the energy calculation is adjusted for MIU, the variations in energy value is exaggerated. Soybean oil energy values ranges from 4220 kcal kg^−1^ to 8920 kcal kg^−1^ for young poultry; compared to the average energy value of analysed samples, the minimum calculated energy had a 45% reduction in energy value. The most homogeneous energy values were reported in rapeseed and linseed oil samples – the minimum calculated values were only 3.0% and 3.4% different from the maximum, and 2.8% and 2.4% from the average.

## DISCUSSION

In general, the results of the current analysis agree with the average of the fatty acid compositions published in the literature for fats and oils commonly used in animal nutrition. However, it is clear from the PCA in Fig. 1 that the prediction of source from fatty acid profiles is difficult and not absolute, given the large overlap in profiles. These effects of cultivar, environment and origin are well documented for vegetable oils.^18^, ^19^ For practical purposes, this indicates that nutritionists formulating to specific fatty acid levels – for example α‐linoleic acid in laying hen diets – cannot fully rely on published values of fatty acid profile but require regular assessment of the oils available to their formulations.

Variation is also present throughout the dataset in terms of the energy values of the fat and oil samples, both within the analysed dataset, and when comparing the current data to published literature values. When MIU is considered using a modified Wiseman equation (Eqn (2)), average energy values can differ substantially from published estimates. Very high levels of MIU may be artefacts of sample collection. For soybean oil, seven samples showed unsaponifiable levels in excess of 100 g kg^−1^, with five samples >250 unsaponifiables. These seven values from a population of 247 individual soybean oil samples are 6–27 times higher than the average of 170 g unsaponifiables^−1^, and likely represent samples taken from less than ideal storage conditions, including the bottom of bulk tanks.

As shown by the correlations between parameters, one of the most influential parameters for assessing or predicting the quality of oils is the level of FFAs in the sample. Higher FFAs are positively correlated with increased MIU presence, increased sensitivity to, and progress of, oxidation and a decrease in the levels of poly‐unsaturated fatty acids (PUFAs). This is supported by the existing literature – FFA have been shown to exert a pro‐oxidant effect,^20^ independently of oil source and existing levels of oxidation, although the ability of FFA to promote lipid oxidation decreases with decreasing pH, especially if the pH is below the pKa value of FFA.^21^ This pro‐oxidant action is likely to be exerted by the carboxylic molecular group, which accelerates the rate of decomposition of hydroperoxides.^22^ However, in this analysis there were fewer significant correlations between the levels of FFA and oxidation than could be expected, especially in the most unsaturated oils. This may partially be due to the variation in FFA levels – levels of FFA in the analysed samples of this dataset are very variable, up to a maximum of 286 g kg^−1^ of oleic acid levels – and may be related to the fate of oxidation products.

High FFA content in oils is unlikely to occur naturally, and may be due to intentional blending of TGs with FFA; if the FFA are from a different oil source to the main lipid source this can affect the fatty acid profile, changing the oil's characteristics and behaviour. The mean FFA content across the dataset is in line with previously reported levels in commercial fat and oil samples: Zumbado *et al*.^23^ reported levels < 10% and Liu *et al*.^24^ reported levels of 0.3% to 3.7%. It may be that the current samples submitted for assessment were those where the quality was doubted, potentially creating a dataset that has an overrepresentation of ‘poor’ quality oils compared to commercial reality – this may also explain the large discrepancy between the mean and median values of analysed FFA contents.

Post‐generation, some FFA oxidation products may end as part of the MIU fraction. Across the dataset, FFA levels are strongly positively correlated with MIU presence in the animal fats. This contrasts with the findings of Liu *et al*.,^24^ who found no significant correlation between FFA and MIU, though there were significantly positive correlations between FFA and the individual measurements of moisture and insolubles. As with the FFA, the levels of MIU seen in the current analysis is high, compared to previous assessments of MIU in fat and oil samples from animal nutrition which reported levels of 0.3% to 1.3%^23^ and 5.8% to 51.6%.^25^

Another potential energy diluting factor is oxidation, as most oils are susceptible to oxidation as a result of their unsaturated fatty acids.^26^ Recent studies have shown that the inclusion of oxidized oils has a negative impact on the growth performance of both pigs and poultry,^27^, ^28^, ^29^ indicating lower energy utilization. In the current study, 0.34% of samples from the ten major lipid sources tested had combined PV and TBA values that indicate early stages of oxidation, with 0.10 of samples showing strong signs of oxidation. Oxidation affects all groups of oils, though some types of oils seem less susceptible; oils with high levels of long‐chain PUFA tend to be more susceptible than those containing higher levels of short‐ or medium‐chain fatty acids.^30^ However, as no historic details were available for the analysed samples, the addition of antioxidants by some lipid producers may be a confounding factor when correlating lipid type and oxidation. Primary lipid oxidation products are not adsorbed, but are not stable and will be broken down by hydrolysis in the gut;^31^ this generates new reactive oxygen species, exerting oxidative stress in the gastro‐intestinal tract.^32^

Interestingly, there were fewer correlations between the measures of oxidation than expected: Liu *et al*.^24^ reported significantly positive correlations between PV and thiobarbituric acid reactive substances (TBARS) levels in an assessment of various vegetable oils subjected to oxidation pressure, though there was no significant correlation between PV and OSI. In the current analysis, there were no significant correlations between PV and TBA values in the dataset. Similarly, PV are not consistently correlated with the OSI measurement of sensitivity to oxidation. There are consistent relationships between the TBA and OSI: in all oils except soybean, significant, negative relationships indicate that high TBA values are correlated to lower OSI values and increased sensitivity to oxidation. To the authors' knowledge, this is the first report expressing these negative correlations between OSI and TBA, albeit weak.

Gray^33^ pointed out that there is no single chemical method which can entirely predict or explain changes in organoleptic properties of oxidized lipids, and the current results highlight the importance of using multiple measures to assess the oxidative status of a lipid sample. The reduction in performance seen with oxidized fats^34^, ^35^ may also be due to a change in the amount of energy diluting factors: across the dataset there are correlations between MIU presence and the level of oxidation; correlations between MIU and TBA are generally positive, though rarely significant. The stronger correlation with TBA values than with PV is likely because the secondary oxidation products are produced more often during industrial manufacture and processing;^36^ the same process will lead to higher levels of FFAs. It may also be that some oxidation products are included in the MIU fraction. FFA and MIU are positively correlated in all oils, with significant relationships reported for all the analysed animal fats and for soya oil.

Relationships between MIU, FFA and oxidation tend to follow similar trends across the dataset, except for the relationship between MIU and OSI. Why MIU presence has differing effects on OSI between lipid sources is not clear. Given that these correlations are driven primarily by significant correlations between OSI and the unsaponifiable fraction, it may be that there are specific molecules in the unsaponifiable fraction of oils where MIU is correlated with high OSI that act in an antioxidant fashion. Further work to identify the unsaponifiable compounds whose presence is more strongly correlated with increased OSI may be interesting for the development of novel antioxidants.

In summary, the assessment of the fat and oil samples conducted in the current analysis highlights the variability of composition and quality of fats and oils available to animal nutritionists. While oils largely conform to published profiles, there is wide diversity of fatty acid composition within oil types; this variation in energy value is greater than is usually assumed by nutritionists. The presence of energy diluting factors is widespread, with the modified energy equation demonstrating the potential energetic loss these compounds incur. Further work should investigate the effect of high levels of MIU on animal performance, validating the accuracy of the adapted Wiseman equation. Similarly, oxidation sensitivity and occurrence is common, though there is limited correlation between the chosen measures of oxidation – likely due to the temporal instability of peroxides – and that one cannot be used to predict the other. Regular assessment of the composition and quality of the oils used in commercial animal nutrition is required for precise and effective feed formulation.
